# Assessing recall bias in post-conflict food security and resilience study in south Wollo zone, Ethiopia

**DOI:** 10.1016/j.mex.2025.103321

**Published:** 2025-04-16

**Authors:** Jemal Hassen Muhyie, Desalegn Yayeh Ayal, Temesgen Tilahun Teshome

**Affiliations:** Center for Food Security Studies, College of Development Studies, Addis Ababa University, Ethiopia

**Keywords:** Conflict, Data analysis, Flashbulb memory test, Food security, Recall bias, Resilience, Flashbulb memory test for recall bias assessment in retrospective data collection

## Abstract

Conflict undermines food security resilience by disrupting the food system while data collection is often constrained during conflict periods despite the need for longitudinal studies for decision making. In such a situation, retrospective data collection that is collected post-conflict is an option. However, recall data lack reliability due to the impact of recall bias. Therefore, statistical methods can be deployed to check reliability of retrospective data for statistical inference. This research integrated a complementary study using flashbulb memory test by including questions regarding a well-known event and assessed recall bias in food security study.

• We collected retrospective data from respondents on household resilience to food insecurity of conflict affected households in South Wollo Zone, Ethiopia.
• By including a set question related to a known event, conflict exposure and control variables (age, gender and education), we demonstrated how the flashbulb memory test can be used to identify potential recall bias.

Conflict exposure and the control variables were not statistically significant on logistic regression for this study which assured that recall bias did not affect the food security and resilience study. We conclude that data can be collected during post-conflict and its statistical reliability can be assessed using the flashbulb memory test and through Common Method Bias (CMB) statistical tests.

## Graphical abstract

**Figure:**
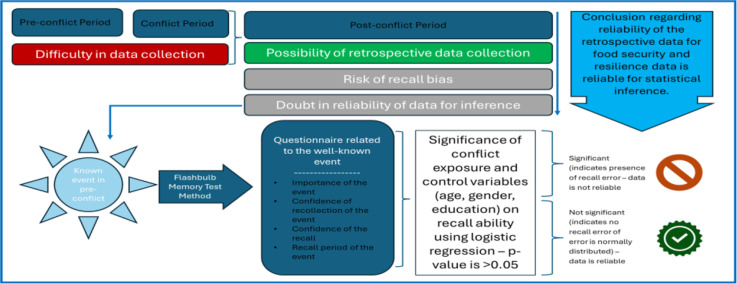

Specifications table

**Table:** 

Subject area:	Food Science
More specific subject area:	Resilience to food insecurity study
Name of your method:	Flashbulb memory test for recall bias assessment in retrospective data collection
Name and reference of original method:	Moreno-Serra, R., Anaya-Montes, M., León-Giraldo, S., & Bernal, O. (2022). Addressing recall bias in (post-) conflict data collection and analysis: lessons from a large-scale health survey in Colombia. Conflict and health, 16(1), 1–14.
Resource availability:	Not applicable

## Background

Conflicts have become more common in developing countries, with devastating and disproportionate impacts on the socio-economic well-being of people as a result of the disruption of economic activities, destruction of basic service infrastructures, causalities, and displacement [[1], [2], [3], [4], [5], [6]]. Assessment of health trends and, well-being is crucial for the development of policies pertaining to conflict-affected communities [7]. Even though it is important to establish trends in data, data collection is constrained by violent conflicts during times of conflict due to access and safety concerns which affects reliability to make statistical inferences [[1], [3]].

In the context of violent conflict, researchers rely on recall information which poses a significant risk to the quality of the data, as recall is affected by several factors [8]. These factors include the types and phrasing of questions, a decrease in the ability to recall as time passes [9,10], erroneous retrieval of events due to negative emotional and mental consequences of the conflict, and deliberately providing incorrect information due to fear.

Despite the method of data collection, reliance on recall data for past events is subject to recall bias owing to inaccuracies or poor recollection of past events by participants. When the recall error is random, the data can be used for inference. However, if a certain group of respondents is particularly affected by recall bias due to certain characteristics, this affects the reliability of the data analysis [[10], [11]].

Researchers collect conflict-related baseline information by asking households or individuals to recall the event and provide data on the impact of conflict on certain socio-economic outcomes, such as health and food security. However, people may not remember all the important events during the conflict period, which affects the quality of the baseline information gathered on a recall basis. In addition to the time factor, recall bias could also be a result of the potential psychological impact of conflict on the mental well-being of affected people, which could create a bias in recalling [8]. Therefore, recall bias is considered a major problem in analysing conflict information collected based on recall [7].

**Fig. 1:**
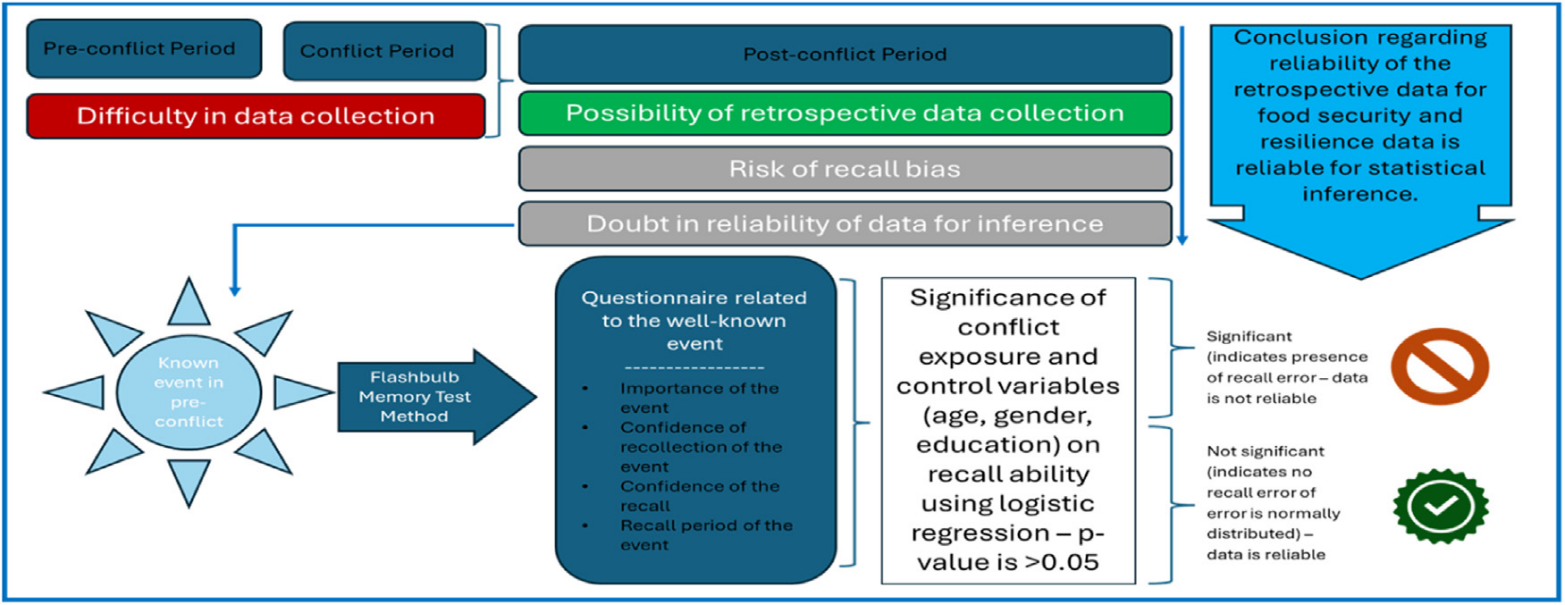
Graphical abstract for flashbulb memory test method: Source: Authors' own construction.

Recall bias cannot be entirely avoided. However, different strategies can be deployed to understand the presence of recall bias and check reliability of data for statistical inference. The study incorporated a complementary analysis using the flashbulb memory test to assess recall bias in the food security and resilience study in South Wollo Zone, Ethiopia. The official announcement of the late Prime Minister Meles Zenawi’s death was used as a reference event to evaluate respondents' recall accuracy. This approach enabled the evaluation of recall bias presence and distribution among respondents, assessing whether recall errors were random and ensuring the reliability of retrospective data for robust statistical inference. The flashbulb memory test method was used to assess recall bias by examining the recollection of significant events by respondents vividly and in detail, serving as a benchmark to evaluate accuracy of retrospective data [12]. The method is relevant in checking consistency of respondents’ memory over a period of time and provides insightful information regarding reliability of data collected on the basis of recall. However, there are limitation of the flashbulb memory test method that should be understood clearly. Despite its vividness, flashbulb memories also suffer inaccuracies and can be affected by personal bias and external factors. In addition, the flashbulb memory test could be more accurate if it is done multiple surveys that require extensive data collection which could be time consuming and expensive [13]. Fig. 1 provides a visual demonstration of how the Flashbulb memory test method works in post-conflict data collection, including guidance on key considerations, statistical approaches, and interpretation of statistical tests.

We integrated the flashbulb memory test, a complementary method into the food security and resilience study, identifies whether recall bias was present or statistically significant, determining data reliability for statistical inference. However, the use of retrospective data is also problematic in general, and it is important to check for the Common Method Bias (CMB) to strengthen the reliability of retrospective data. CMB arises when variance in study results is attributable to the measurement method rather than the actual constructs of interest. CMB occurs due to factors such as reliance on the same respondents, uniform question formats, or consistent timing in data collection, leading to inflated relationships between variables [14]. CMB should be tested on the actual study variables, not the recall bias regression model. CMB should be identified and appropriately assessed to increase the validity of statistical conclusions from retrospective data. MacKenzie and Podsakoff [15] indicated that identification and mitigation of CMB helps to deal with unrealistic correlations between variables that might result from response biases. Therefore, employing such a statistical method increases the credibility of empirical results and confirms the robustness of research conclusions.

## Method details

### Description of the study area

This research was conducted in the South Wollo zone of the Amhara region, Ethiopia. The study encompasses a collection of recall data for food security and resilience studies of households affected by drought and conflict. The recall periods were twelve-months period for the pre-conflict (June 2019 to May 2020) and post-conflict reference periods (June 2021 to May 2022).

### Sampling techniques

The selection of the four districts where the food security and resilience study conducted were selected in consultation with key informants considering prior history of exposure to drought and the northern Ethiopia conflict. However, the selection of villages and survey participants followed randomization. A profiling of household information was developed for the four districts in each village. In the four districts, 38 villages were selected on a lottery basis. Simple random sampling was used to identify the participants in the questionnaire-based survey. A list of households was developed for all residents of the 38 randomly selected villages. A quota of samples was assigned to each village based on the total number of samples determined as indicated below. Then, a systematic random sampling was deployed to identify survey respondents proportional to the population of each village randomly. This approach helped to ensure representative of survey respondents. The sample size was determined based on Taherdoost [16] at a 95 % confidence interval with a 5 % margin of error with 10 % contingency included, which makes the total sample 422, where n is the sample size and p is the percentage of occurrence of state estimated at 50 % to produce the maximum sample size [17].

$$n=\frac{p\left(100-p\right)z^{2}}{e^{2}}$$

$$n=\frac{0.5\left(1-0.5\right)1.96^{2}}{0.05^{2}}=384$$

### Data sources

Primary data were collected through a questionnaire-based survey of 422 households (15 % were female-headed households). The recall bias questions were part of a questionnaire-based survey that was administered to collect recall data on socio-economic situation, food security status, and resilience of drought- and conflict-affected households. In addition to the household survey, secondary data were collected as part of a review of related literature.

### Techniques of data analysis

The flashbulb memory test was used to analyse recall bias in food security and resilience studies. Moreno-Serra et al. [8] argued that ex-post health surveys that collect recall data from conflict-affected populations are reliable for empirical inference using standard regression methods with the Flashbulb test, which was developed in the fields of psychology and psychometry. In this research, the same method as that used by Moreno-Serra et al. [8] was used for food security and resilience studies. The empirical specification of conflict as adapted to reflect the food security outcome, is as follows.

$$Y_{i}=\alpha_{0}+\alpha_{1}D_{i}+\mu_{i}$$

where $Y_{i}$ is the food security outcome for household i, $\alpha_{0}\;$is the intercept that measures the impact of conflict on the outcome variable, Di is the conflict exposure of household i which takes the value of 1 if exposed or 0 if the household is not exposed to conflict. The general understanding is that in communities affected by conflict, all households are considered to be exposed to conflict impacts and $\mu_{i}\;$is the sum of all errors.

When retrospective data are used in a survey, there could be recall errors, which might be due to memory issues and the negative impact of conflict on people’s ability to recall past events [8]. This recall error affects the estimation of $\alpha_{1}.\;$The interaction is indicated mathematically by the formula below:

$$\mu_{i}=\omega_{i}+\varepsilon_{i}$$

where $\omega_{i}\;$is the survey recall error, and $\varepsilon_{i}$ takes all other errors (observed and unobserved) that are considered random. Conflict exposure is correlated with household characteristics, which results in a more realistic model of recall bias.

$$Y_{i}=\alpha_{0}+\alpha_{1}D_{i}+X_{i}^{{}^{\prime }}a_{x}+\omega_{i}+\varepsilon_{i}$$

In this model with covariates, the condition required for recall errors not to bias $\alpha_{1}$ is that the conditional independence assumption cov (Di|Xi, $\omega_{i}$) = 0 holds in the data. This means that recall errors are randomly distributed across conflict and non-conflict areas once observable characteristics are controlled for.

Brown and Kulik [18] suggested a complementary model to understand recall bias, called the flashbulb test, which suggests that major events such as natural tragedies or traumatic events can be recalled through the use of well-known events and objectively corroborated. X is the presence of recall bias computed using pre- and post-conflict scenarios, as a comparison between two times is necessary.

$$\omega_{i}=\left\{ \begin{array}{c} 0\;if\;Z_{t-1}=\hat{Z}t-1,\text{by}\;\text{individual}\;i\;\text{at}\;\text{the}\;\text{time}\;\text{of}\;\text{the}\;\text{post}-\text{conflict}\;\text{survey}\;t\\ 1\;if\;Z_{t-1}\neq \hat{Z}t-1,\text{by}\;\text{individual}\;i\;\text{at}\;\text{the}\;\text{time}\;\text{of}\;\text{the}\;\text{post}-\text{conflict}\;\text{survey}\;t \end{array}\right.$$

where $Z_{t-1}$ is a well-known past event occurred at time “*t* − 1” (pre-conflict) and $\hat{Z}t-1$, it denotes the recall of the event by individual i at the time of the post-conflict survey “t”. We can then test the conditional independence assumption in a model with covariates cov (Di|Xi, $\omega_{i}$) = 0 by estimating the following regression:

$$\omega_{i}=\beta_{0}+\beta_{1}D_{i}+X_{i}^{{}^{\prime }}\beta_{x}+\varphi_{i}$$

where $\omega_{i}$ ∈ {0, 1} is an indicator that takes the value of zero if individual i accurately retrieves the past event (recall accuracy by comparing responses of respondents to the actual timing of the event), and 1 if the individual retrieves the event inaccurately, $\beta_{0}$ is the intercept, Di ∈ {0, 1} is an indicator that takes the value of 1 for conflict-affected and 0 otherwise, Xi contains the control variables, including age of the household head, education in number of years of the household head, gender of the household head, and $\varphi_{i}\;$is the error term.

For the flashbulb memory test, this research considers the official announcement of the death of the late Prime Minister of Ethiopia, Mr. Meles Zenawi. Respondents were asked to check if they recalled the event, and statistical inference was made as a proxy to determine the presence of recall bias in the food security and resilience study. A questionnaire was designed to assess the clarity of the recall, importance of the event, accuracy of the recall against known timelines, level of confidence of respondents on their recollection of the event, and their recall.

The questionnaire was an integral part of the food security and resilience survey and was administered along with the main research undertaking. Stata was used as the analysis software while conducting the analysis using appropriate statistical approaches.

Other control variables that did not often change because of exposure to conflict were included in the logistic regression model. Data were collected as an integral part of the food security and resilience survey using the following questions for recall bias analysis (Table 1).

**Table 1:** Flashbulb recall test questionnaire.

S/N	Recall questions	Response options
1	How clearly you recall the official announcement of the death of the late prime minister Mr. Meles Zenawi of Ethiopia?	1. Does not recall it 2. Recalls it vaguely 3. Recalls it more or less clearly 4. Recalls it very clearly 5. Recalls it vividly
2	At that time, how much importance did you attach to the event?	1. Not important at all 2. Less important to me 3. Somewhat important 4. Considered it very important 5. Considered it extremely important
3	What was the year and month of the official announcement of his death?	1. August 2011 2. August 2012 3. August 2013 4. January 2011 5. January 2012 6. January 2013
4	On a scale of 1 to 5 (where 1 is not at all confident and 5 is extremely confident), how confident do you feel about this remembrance?	1. Not confident at all 2. Less confident 3. Moderately confident 4. Very confident 5. Extremely confident

*Source:* Researchers’ construction.

Furthermore, several statistical techniques are available to detect CMB in Stata, including Harman’s Single-Factor Test, the Marker Variable Technique, and the Correlation Matrix Approach.

**Harman’s Single-Factor Test:** One of the most commonly used techniques, Harman’s Single-Factor Test, assesses whether a single factor accounts for most of the variance in the dataset. If one factor explains >50 % of the total variance, CMB may be present [14]. The mathematical formulation of this test is based on exploratory factor analysis (EFA), where the total variance explained by the first factor is computed as:

$$\lambda_{1}/\sum \lambda_{i}$$

where:

$\lambda_{1}$ is the eigenvalue of the first unrotated factor,
$\sum \lambda_{i}$ is the sum of all eigenvalues.

*Interpretation of Harman’s Single-Factor Test*: If the first factor accounts for >50 % of the variance, it indicates potential CMB and suggests that additional remedies may be required.

**Marker Variable Technique:** This technique introduces a theoretically unrelated variable, known as a marker variable, to detect potential method bias. If the inclusion of the marker variable significantly alters regression coefficients, CMB is likely influencing the results [19]. The statistical formulation of this test is:

$$Y=\beta_{0}+\beta_{1}X+\beta_{2}MV+\varepsilon$$

where:

Y is the dependent variable,
X represents the independent variables,
MV is the marker variable,
$\beta_{2}$ captures the effect of the marker variable,
ε is the error term

*Interpretation of the Marker Variable Technique*: A significant change in the coefficients after including the marker variable suggests the presence of CMB.

**Correlation Matrix Approach (Full Collinearity Test):** High correlations among independent variables, particularly above 0.90, indicate the possibility of CMB. The mathematical formulation for detecting multicollinearity using a correlation matrix is:

$$\rho_{ij}=\frac{Cov\left(X_{i},X_{J}\right)\;}{\sigma x_{i}\sigma x_{j}}$$

where:

$\rho_{ij}$ is the Pearson correlation coefficient between variables and $X_{i}\;and\;X_{J}$,
$Cov(X_{i},X_{J})\;$is the covariance between the two variables,
$\sigma x_{i}\;and\;\sigma x_{j}$ and are the standard deviations of the respective variables.

*Interpretation of Correlation Matrix Approach (Full Collinearity Test):* If correlations exceed 0.90, it suggests multicollinearity that may be driven by common method variance.

## Method validation

### Demographic characteristics of respondents

Among the respondents, 15 % were female-headed and 85 % were male-headed households from whom data were collected (Table 2).

**Table 2:** Number of samples per study district and gender of the household head.

Name of study districts	# of observations			Proportion of response ( %)
	Male-headed households	Female-headed households	Total number of households	
**Delanta**	72	17	89	21
**Dessie Zuria**	123	15	138	33
**Kalu**	77	11	88	21
**Werebabo**	86	21	107	25
**Total**	358	64	422	100

*Source:* Survey data by researchers.

Respondents reported on their perceived exposure and severity of the conflict damage on the overall food system that affects household food security. Only 10 % reported that their food security was not affected by the conflict, and 19 %, 25 %, and 46 % of respondents indicated mild, moderate, and severe damage, respectively (Table 3).

**Table 3:** Level or severity of conflict impact on food system related to household food security.

Rate of conflict impact on food system	# of observations	Proportion of response ( %)
No damage	43	10
Mild damage	79	19
Moderate damage	107	25
Severe damage	193	46
Total	422	100

*Source:* Survey data by researchers.

The minimum age range from 20 years and the maximum age of the household head was 79 years. Generally, the majority of respondents fell within 30 – 49 years representing a cumulative proportion of >61 % (Table 4).

**Table 4:** Age categories of household heads and proportion of respondents.

Age categories of household heads	# of observations	Proportion of response ( %)
20 – 29 years	13	3.1
30 – 39 years	102	24.2
40 – 49 years	158	37.4
50 – 59 years	95	23.5
60 – 69 years	44	10.4
70 – 79 years	10	2.4
Total	422	100

*Source:* Survey data by researchers.

In the study areas, only 30 percent of respondents reported practicing off farm activities for income generation in the post-conflict context with labour activities represent the bigger proportion. The conflict negatively affected livelihood diversification that limits ability of households to access food at household level.

Based on the flashbulb memory test questionnaire designed for the study, respondents were asked questions to check the clarity of their recall of the official announcement of the death of the former Prime Minister of Ethiopia. The question was intended to check whether the respondents clearly recalled the event (Table 5).

**Table 5:** Clarity of the recall of the event by respondents' answer.

Clarity of recall	# of observations	Proportion of response ( %)
Does not recall it	105	24.9
Recalls it vaguely	106	25.1
Recalls it more or less clearly	92	21.8
Recalls it very clearly	55	13.0
Recalls it vividly	64	15.2
Total	422	100

*Source:* Survey data by researchers.

In addition, we checked the amount of value or importance that respondents attached to the event. The importance of the event is linked to weather respondents being interested in the event due to its national and political dynamics, and whether the event was personally relevant (Table 6).

**Table 6:** Importance or relevance of the recall of the event by respondents' answer.

Importance of recall	# of observations	Proportion of response ( %)
Not important at all	90	21.3
Less important to me	199	47.2
Somewhat important	59	14.0
Considered it very important	39	9.2
Considered it extremely important	35	8.3
Total	422	100

*Source:* Survey data by researchers.

Finally, the respondents were asked to indicate the exact period of the event. Considering that the data collection occurred in the rural context of Ethiopia, the researchers opted to ask for the year and month of the event instead of asking for the exact date of the official announcement of the death of the late Prime Minister of Ethiopia, Mr. Meles Zenawi. Responses were provided to the respondents to assist in the better recollection of the event (Table 7).

**Table 7:** Exact period of the event by respondents' answer.

Exact period of the event	# of observations	Proportion of response ( %)
August 2011	19	4.5
August 2012	297	70.4
August 2013	29	6.9
January 2011	27	6.4
January 2012	30	7.1
January 2013	20	4.7
Total	422	100

Source: Survey data by researchers.

Further to the recall of the event as indicated above, respondents were asked how confident they felt in the recollection of the event and in the response, they provided to the confidence question (Table 8).

**Table 8:** Respondents' confidence on the recollection of the event.

Confidence level of the recall	# of observations	Proportion of response ( %)
Not confident at all	94	22.3
Less confident	111	26.3
Moderately confident	86	20.4
Very confident	60	14.2
Extremely confident	71	16.8
Total	422	100

Source: Survey data by researchers.

The question related to the exact period of the event (in terms of year and month) was used to determine how many of the respondents recalled the event correctly. The table below indicates that 70 % of respondents recalled the event correctly in August 2012, while 30 % received the recall incorrectly (Table 9).

**Table 9:** Proportion of respondents and accuracy of recall.

Correctness of recall	# of observations	Proportion of response ( %)
Correct recall	297	70
Incorrect recall	125	30
Total	422	100

Source: Survey data by researchers.

Based on the correctness of the recall of the event, the responses were categorized into two groups for the flashbulb memory test. Respondents who recalled the event correctly were assigned 0, while respondents who recalled the event incorrectly were assigned 1, which denotes the recall error or bias. This category forms the recall error as an outcome variable, and logistic regression is used to identify factors with or without recall bias.

### Recall bias and association with socio-economic and demographic characteristics

A chi-square test was run to determine any significant association between recall error and different socioeconomic and demographic characteristics of the respondents. The socioeconomic and demographic characteristics included district (Pr = 0.880), sex of household head (Pr = 0.544), marital status (Pr = 0.879), conflict exposure (Pr = 0.863), severity of conflict impact (Pr = 0.997), food security status on an HFIAS scale (Pr = 0.257), age of the household head (Pr = 0.495), agroecology (Pr = 0.584), off-farm participation (Pr = 0.314), and food security status on the household food balance model (Pr = 0.284). The chi-square test indicated that none of the socioeconomic or demographic characteristics were significantly associated with the ability of respondents to recall past events.

### Flashbulb memory test

A logistic regression model was used for the recall error as a binary dependent variable, taking a value of 0 for correct recall and 1 for incorrect recall recalls. The level of importance that respondents attached to the event, the rate of conflict damage on food systems, and its impact on household food security as a categorical variable, and control variables such as gender, age, and years of education of the household head were the independent variables included in the regression model. The inclusion of gender, age and education of the household were identified as key control variables for the study. Gender plays a crucial role in memory and emotional connection which can affect memory ability differently [12] particularly in conflict setting. Besides memory varies with age and cognitive ability could decline as age increases [13]. Education also plays a crucial role in processing of information and memory retention with educated individuals may recall events accurately due to cognitive differences. In the study, other socioeconomic and demographic factors were excluded due to their indirect influence on memory. Income, family size or occupation could affect stress and improve wellbeing that could relate with memory ability. However, it is not strong and often indirect in terms of affecting memory ability. Therefore, we recommended to include age, gender and education status of the household head who provided the response during the survey as control variables.

A logit model was constructed for the two sets of predictor variables. The first set of variables was the level of importance that respondents attached to the event and the rate of conflict damage on food systems and its impact on household food security as a categorical variable (no damage, mild damage, moderate damage, and severe damage) without using any of the control variables. As indicated in the table below, only the importance of the event was a significant predictor of recall error at a p-value of <0.05. However, conflict exposure in general and specifically intensity of conflict expressed in terms of severity of damage to food systems with implications on household food security were found to be insignificant (Table 10).

**Table 10:** Logistic regression of recall error with importance of the event and impact of conflict.

Recall error	Coefficient	Standard Error	z	*P*>|z|	Confidence Interval (95 %)	
Recall importance	−1.559	0.156	−10.0	0.000*	−1.865	−1.254
Conflict exposure						
Mild	0.168	0.533	0.32	0.753	−0.877	1.213
Moderate	0.258	0.519	0.50	0.619	−0.759	1.276
Severe	0.072	0.475	0.15	0.880	−0.859	1.003
**_cons**	4.715	0.589	8.00	0.000*	3.559	5.870

⁎ Significant predictors at 95 % confidence interval, Number of observations is 422; LR Chi^2^=181.47; Prob > chi^2^ = 0.0000 and Pseud R^2^ = 0.3539; Source: Survey data by researchers.

The second option was running the logistic regression by including the control variables of age, gender, and education of the household head, which did not change due to exposure to conflict impacts. While including the control variables in the model, recall importance and the level of conflict’s impact on the food system were kept in the regression process (Table 11).

**Table 11:** Logistic regression of recall error including the control variables.

Recall error	Coefficient	Std. Error⁎⁎	z	*P*>|z|	Confidence Interval (95 %)	
Recall importance	−1.565	0.156	−10.02	0.000*	−1.871	−1.259
Conflict exposure						
Mild	0.114	0.538	0.21	0.832	−0.941	1.169
Moderate	0.213	0.523	0.41	0.684	−0.812	1.237
Severe	0.045	0.477	0.10	0.924	−0.890	0.981
Gender	−0.230	0.382	−0.60	0.547	−0.979	0.519
Age	0.008	0.014	0.54	0.593	−0.020	0.035
Years of education	0.025	0.043	0.59	0.558	−0.059	0.109
**_cons**	4.593	1.054	4.36	0.000*	2.527	6.658

⁎ Significant predictors at 95 % confidence interval, Number of observations is 422; LR Chi^2^= 182.52; Prob > chi^2^ = 0.0000 and Pseud R^2^ = 0.3559,.

⁎⁎ Std. Error is Standard error; Source: Survey data by researchers.

In the logit model, only the recall importance was a significant predictor. Neither conflict impact nor any of the control variables were significant, as the p-value for all was greater than 0.05. As a result, conflict exposure did not affect recall ability, or at least, the pattern of recall bias was normally distributed. This implies that either conflict did not affect recall or that the impact of conflict is random, which does not affect the use of data, considering that there is no pattern in recall ability for any segment of the respondents. The results of the recall bias analysis based on the flashbulb memory test method indicated that the food security and resilience data were not affected by recall bias. Hence, inferences can be made using data from the food security and resilience analysis in the context of conflict exposure in the South Wollo Zone.

### How the finding of the flashbulb memory test compares to other studies

The finding of our study indicates that conflict did not affect recall ability in the context of the study population. However, it also contradicts with existing evidence where conflict affected recall ability that leads to recall bias. For instance, Moreno-Serra et al. [8] utilized a flashbulb memory test to check recall accuracy. They found out that individuals in conflict affected area tend to overstate past situation particularly economic conditions and suffering. This indicates that traumatic experience due to conflict exposure could affect memory ability, leading to overstating or understanding pre-conflict situations. However, these biases were not universal across all types of questions or across all demographic groups highlighting the complexity of recall bias in conflict settings, suggesting that while conflict exposure may lead to certain memory distortions, it does not always result in a systematic over-reporting or under-reporting of all experiences Moreno-Serra et al. [8]. The study emphasizes the need for careful consideration of these biases when analysing retrospective data in conflict-affected populations. In addition to traumatic experience, other factor like political situation, media, humanitarian aid could also determine recall bias.

Our finding indicated that recall ability could be stable even in conflict context. A study by De Waal [20] indicated that communities that are affected by recurrent food insecurity develop recollection mechanisms that help them in accurately remembering retrospective information. remember past events. This finding could be related to the context of South Wollo Zone. South Wollo zone is considered as a ‘famine belt’ of Ethiopia due to recurrent exposure to drought. People in South Wollo are exposed to several climate-induced calamities including drought that might help in developing such recollection strategies which should be further studied to finetune the finding of the study.

In addition, demographic characteristics like age, gender, and education were not found significant where this finding differs from [21]) who reported that gender, age and education affects recall ability. The difference in the finding of these studies could be attributed to type of questions, intensity of the conflict impact and other socioeconomic factors.

We argue that generalizing similar studies may be problematic as understanding of recall bias in conflict setting requires a context-specific assessment. While some research indicates systematic distortions in retrospective self-reports due to traumatic events, our findings suggest that, at least for food security and resilience assessment in the study areas, recall remains relatively accurate across various demographic groups this affirms the reliability of the recall data for statistical inference. This underscores the importance of not generalizing recall bias effects and highlights the need for tailored methodologies when assessing recall accuracy in diverse conflict-affected populations by incorporating a complementary approach, like the flashbulb memory test method in food security and resilience study and appropriate actions should be taken before deciding to use data for statistical inference.

### Testing common method bias

**Fig. 2:**
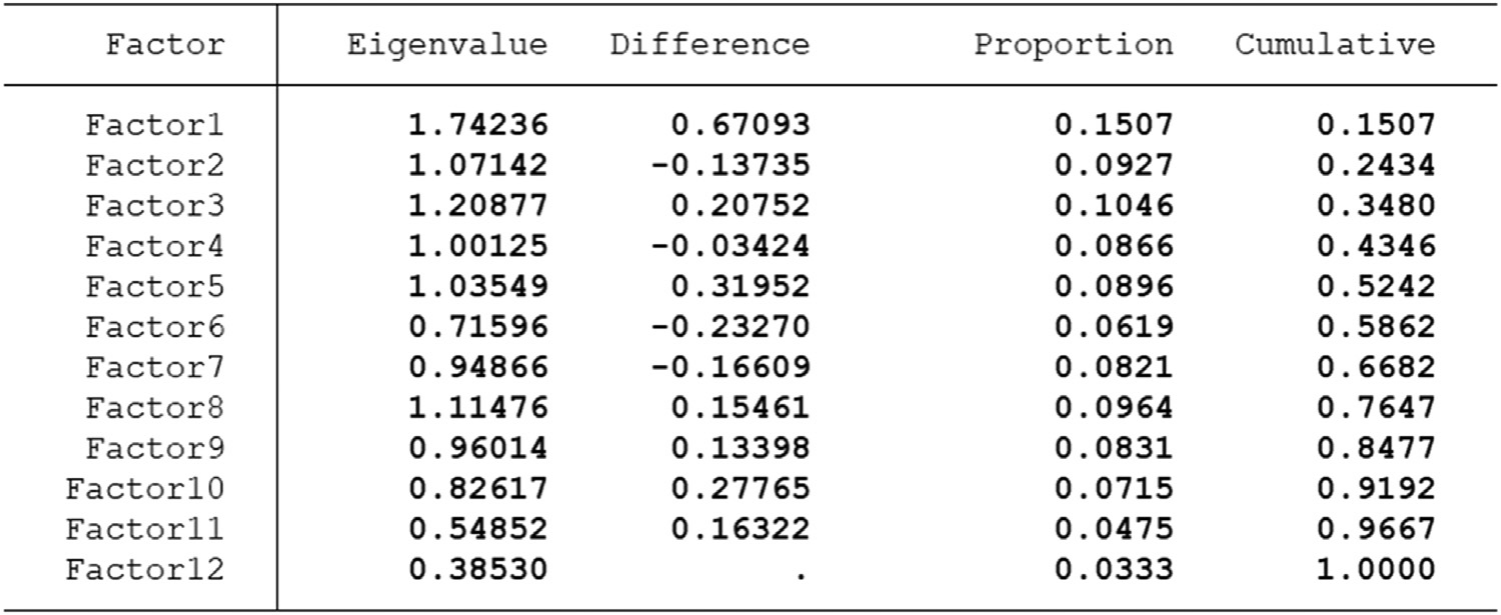
Harman's Single-Factor Test; Source: Survey data. # of observation = 422, retained factors = 12, # of params = 150, Schwarz's BIC = 907.14 and Akaike's) AIC = 300.389.

The Common Method Bias test was conducted by deploying a combination different statistical methods: Harman’s Single-Factor Test, Marker Variable Technique and Correlation Matrix Approach to strengthen statistical validity of research findings for inference (Fig. 2).

As the first factor accounts for <50 % of the variance, it indicates that Common Method Bias (CMB) is not a problem for the data. The Marker Variable Technique also determined that CMB is not a problem for the retrospective food security and resilience analysis. The researchers selected a factor variable called woreda (which is district). The p-value for woreda was 0.130 which is greater than 0.05 confirming it is not significant meaning CMB is not a problem for the retrospective data. Furthermore, the multicollinearity test for the independent variables indicated that there are no variables that are highly correlated. In the statistical test, multicollinearity which could be a result of CMB was not detected. The maximum correlation between independent variable was 0.48 which is not high to determine presence of CMB. The Variance Inflation Factor (VIF) was also <3 for all independent variables indicating no serious multicollinearity affirming that CMB is not a problem for the retrospective study in our study context.

## Conclusion and recommendations

Recall bias assessment and analysis were conducted as a complementary study to the study of household food security and resilience of drought- and conflict-affected households in the South Wollo Zone. Following the Northern Ethiopian conflict, which devastated thousands of households in the zone, a food security and resilience assessment was conducted in four districts: Delanta, Dessie Zuria, Kalu, and Werebabo.

This study aimed to compare household resilience to food insecurity during pre-conflict and post-conflict reference periods, taking into account one year before the Northern Ethiopian conflict as the baseline, one-year periods of the conflict, and after the peace agreement as the follow-up period. Owing to the ongoing conflict, it was not possible to collect real-time data for the conflict period, and there were no available data for pre-conflict reference. As a result, it was necessary to collect retrospective data for the two reference periods based on the respondents’ recall.

In the context of conflict, making comparisons before, during, and after the conflict is crucial for developing policies pertaining to conflict-affected communities. The challenges of access and safety concerns make data collection during conflict periods very difficult. As a result, recalling information was the most appropriate option. However, recall data suffer from reliability and credibility due to potential errors due to loss of memory or forgetfulness, erroneous retrieval of past events, psychological consequences of the conflict, or deliberate misinformation due to fear. Reliability makes it difficult to make statistical inferences with the expected level of confidence.

To minimize recall bias, researchers are recommended to conduct research that covers a short time span to guarantee proper retrieval of past events. In addition, the design of surveys should also consider the potential for recall bias that affects the credibility of research recommendations. However, there are times in the case of this research where data collection was not possible during the conflict and there is a need to make a comparison between two reference periods to understand the impact of conflict on food security, livelihoods, and resilience mechanisms. The decision to use recall data is imperative.

While testing the presence of recall bias using methods like flashbulb memory test to determine statistical reliability of inferences, it is important that researchers should deploy different strategies to reduce recall bias in retrospective data collection. In this study, we implemented different strategies during the design of surveys and at time of data collection to ensure accurate data was collected. These strategies include the use of seasonal reference during production related questions in food security study. The major agricultural seasons in the study are include *belg* and *meher*. These agricultural seasons were used as reference to collect agricultural production and disaster exposure related questions in the study to help respondents accurately recall such information.

The other aspect was related to the selection of enumerators and the quality of the training on the data collection tools that was provided to enumerators. The researchers identified agricultural experts who knows the local context and who have understanding of food security, disaster risk reduction and resilience. This was coupled with a comprehensive training on the household questionnaire which enhanced their understanding. The training also included interviewing skills to ensure some information could be verified and triangulated for accuracy. The allocation of sufficient amount of time for data collection was another strategy that was utilized. This helped enumerators to collect few questionnaires everyday and allocate sufficient amount of time when dealing with old people and people who require support during the interviews. Despite the financial burden of this strategy, it was found to be relevant considering the cost of rushing would be even bigger as it results in poor quality data.

Therefore, in the design of surveys, it is recommended to include a well-known event and assess whether respondents are able to recall it. The questionnaire also included questions related to the clarity of recall, importance of the event, actual timing of the event, and confidence of respondents in the recollection of the event. The recall error, an outcome variable, is generated, and a logistic regression is run using predictors such as recall importance and intensity of conflict to understand whether conflict exposure was a significant predictor of recall bias. Furthermore, control variables such as age, education, and gender of the household heads were included in the model. The regression outcome indicated that conflict exposure, expressed as the intensity of conflict in the food system, an indicator of household food security, was not significant. The research concluded that recall bias does not affect food security and resilience. Hence, the statistical inferences were valid and reliable as they were not influenced by recall bias.

The finding of the flashbulb memory test could be generalized to other conflict-affected contexts if there are similarities in conflict dynamics, population characteristics, and other socio-economic factors to consider the observed patterns in the study that may be applicable in other contexts. However, it is important to note that there are limitations in generalizing the findings as memory ability could be affected by different factors such as differences in cultural and social elements, conflict intensity, the influence of media, and the representativeness of samples. Therefore, future studies should replicate the methodology in diverse conflict settings to strengthen the external validity of the results. This study underscores the importance of not generalizing recall bias effects and highlights the need for tailored methodologies when assessing recall accuracy in diverse conflict-affected populations by incorporating a complementary approach, like the flashbulb memory test method in food security and resilience study and appropriate actions should be taken before deciding to use data for statistical inference.

Finally, we recommend complementing the flashbulb memory test method, we recommend that other statistical methods like Harman’s Single-Factor Test, Marker Variable Technique, and Correlation Matrix Approach can be deployed to assess the presence of Common Method Bias (CMB), which is a major problem in a retrospective study. This approach will help identify issues, address them using the statistical method, and confirm the validity and rigor of statistical inference in retrospective food security and resilience studies.

## Limitations

Not applicable.

## Ethics statements

The Institutional Review Board (IRB) of Addis Ababa University approved all data collection tools and processes. Ethical clearance was provided to the researchers following institutional review board approval. In addition, the objective of the research was clarified to the participants, and verbal consent was obtained before the start of the interviews. The entire research process, including data collection, analysis, storage, and communication, maintains confidentiality and ethical principles.

## CRediT author statement

**Jemal Hassen Muhyie:** contributed to the study design, data collection, analysis, and writing of the manuscript. **Desalegn Yayeh Ayal (PhD) and Temesgen Tilahun Teshome (PhD):** contributed to the design, analysis, and review of this manuscript.

## Declaration of competing interest

The authors declare that they have no known competing financial interests or personal relationships that could have appeared to influence the work reported in this paper.

## Data Availability

Data will be made available on request.
